# Determination of the Heavy Metal Bioaccumulation Patterns in Muscles of Two Species of Mullets from the Southern Caspian Sea

**DOI:** 10.3390/ani12202819

**Published:** 2022-10-18

**Authors:** Shima Bakhshalizadeh, Rafael Mora-Medina, Francesco Fazio, Vincenzo Parrino, Nahúm Ayala-Soldado

**Affiliations:** 1 Department of Marine Science, Caspian Sea Basin Research Center, University of Guilan, Rasht 41335-1914, Iran; 2 Department of Anatomy and Comparative Pathology and Toxicology, Faculty of Veterinary Medicine, University of Córdoba, 14071 Córdoba, Spain; 3 Department of Veterinary Science, University of Messina, 98122 Messina, Italy; 4 Department of Chemical, Biological, Pharmaceutical and Environmental Sciences, University of Messina, 98122 Messina, Italy

**Keywords:** fish muscles, health risk, pollution, trace metals

## Abstract

**Simple Summary:**

Fish are an important source of proteins of a high biological value, of some vitamins and minerals, and of polyunsaturated omega-3 fatty acids. However, fish products can also allow harmful substances, like heavy metals, to enter the diet. Such substances are recognized as being the most serious contaminants for aquatic ecosystems at the present time. Their non-biodegradability allows them to accumulate in fish tissues, and then pass into human diets. Therefore, our study aimed to determine the concentrations of heavy metals (As, Cd, Cu, Hg, Ni, Pb, and Zn), and to evaluate the bioaccumulation patterns in the different types of musculature in two species of fish of commercial interest, *Chelon auratus* and *Chelon saliens*, from the south coast of the Caspian Sea. The results obtained emphasize the need to continue to monitor and evaluate the degree of pollution in the sampled area, both in fish and other species and also in the environment, as well as recommending prevention measures orientated towards limiting and/or reducing the excessive exposure of the human population to heavy metal contamination.

**Abstract:**

Although fish is a food that supplies nutrients of a high biological value, they can also be a source of some harmful substances, such as heavy metals. In the same context, some human activities in the Caspian Sea have contaminated this ecosystem during the past few years. For those reasons, our objective consisted of determining the concentrations of heavy metals and evaluating their bioaccumulation patterns in the different types of musculature in two species of mullets of commercial interest, *Chelon auratus* and *Chelon saliens*, from the southern coast of this sea. For this purpose, 20 *C. auratus* and 29 *C. saliens* were caught off this coastline and the metal concentrations in 3 different muscle locations were analyzed: the ventral, dorsal and caudal muscles of each fish. The caudal muscle had higher concentrations of Cd, Cu, Pb, and Zn, whereas As, Hg and Ni accumulation seemed to be independent of the musculature type. Overall, the Cd, Hg, and Pb concentrations exceeded the maximum levels permitted in fish by the European Union. In addition, the relationships between pairs of metals were positive and elevated in all the cases, which could be a sign of heavy metal pollution in the region sampled. Therefore, it will be necessary to continue monitoring and evaluating the degree of pollution in the Caspian Sea.

## 1. Introduction

Fish are an important source of proteins of a high biological value, of some vitamins and minerals, and of polyunsaturated omega-3 fatty acids for the human diet. However, fish products can also enable harmful substances, such as heavy metals, to enter the diet. These contaminants are recognized as being the most serious for aquatic ecosystems at the present time, due to their anthropogenic activity. Their non-biodegradability allows them to accumulate in fish tissues, and then into the human diet [1,2,3,4]. Thus, in the past few years, there has been debate on the benefits and risks of fish consumption that has caused some confusion as to the amount that can be consumed or, even, whether it is recommendable to do so at all [5].

Some essential metals, such as copper (Cu) and zinc (Zn), are necessary for the growth and metabolism of living organisms, and nickel (Ni) is might possibly have a similar role, but no strong evidence exists of this being so as yet [6]. In the case of Zn, a deficiency in the diet triggers dermal problems, and retardation of growth and sexual maturation [7]. Cu deficiency causes arterial weakness, liver problems, and anemia. Although these metals are essential, fish can accumulate them and when their concentrations increase and exceed the toxicity threshold, they can be potentially toxic to humans [8].

Other heavy metals such as arsenic (As), cadmium (Cd), mercury (Hg), and lead (Pb) are known to be non-essential to human health, and in addition to being toxic for aquatic organisms, can also be a problem for human health at very low concentrations [8,9]. For instance, As is classified within group 1 by the International Agency for Research on Cancer (IARC) [10] as carcinogenic in humans. It has been reported that the consumption of water with a high content of inorganic As results in a greater mortality from various types of cancer [11]. Cd can cause osteoporosis, anemia, non-hypertrophic emphysema, irreversible renal tubular lesions, eosinophilia, anosmia, and chronic rhinitis [12]. The adverse effects of both organic and inorganic Hg include cytotoxicity, neurotoxicity, teratogenicity, nephrotoxicity, and immunotoxicity [13,14]. Chronic exposure to Pb can cause brain damage, psychosis, autism, and dyslexia, among other pathologies [15]. Due to the high toxicity of these elements, water pollution is a very important issue for wildlife and human health.

The Caspian Sea, with a surface of approximately 370,000 km^2^, is the largest closed sea in the world. It is located between Asia and Europe, and is bordered by five countries: Russia, Kazakhstan, Turkmenistan, Iran, and Azerbaijan. In the past few decades, this ecosystem has increasingly suffered from climate factors, with fluctuations in its water level and salinity variations, and also changes due to human activities, such as mining, oil drilling, and over-exploitation of fishing [16]. Due to the fact that heavy metals can reach aquatic environments by means of both geogenic and anthropogenic sources [17], the degree of pollution can be highly variable, depending on the area.

The Mugilidae family, whose species are commonly known as mullets, represents a large taxon of coastal marine fish with a worldwide distribution [18]. These fish can spend part of their life cycle in coastal lagoons, lakes, and rivers to rest and mature, and later migrate towards the sea [19]. In the Caspian Sea, mullets were not naturally present. However, *Mugil cephalus*, the most widespread species among the family Mugilidae, was successfully introduced to this region [20] from the Black Sea in the 1930s [21]. Mullets are fished both for food purposes and for aquaculture production, thus these species have a high value [18].

Currently, the information available on the accumulation of metals in the different muscle tissues of fish is limited [22,23]. The muscle system of a teleostean fish is not uniform but is basically composed of two types: red and white [24,25]. The red myotomal muscle fibers in most fish are arranged as being parallel to the longitudinal axis of the body in one or more surface bands, while the white fibers constitute most of the musculature and follow complex helicoidal trajectories in successive myotomes [26,27]. There is a third type of muscle in some species, called intermediate or pink. Their fibers are distributed between the red and white muscles and have intermediate properties [23].

Therefore, our study aimed to determine the concentrations of heavy metals (As, Cd, Cu, Hg, Ni, Pb and Zn), and to evaluate the bioaccumulation patterns in the different types of musculature (white and red muscles) in two species of mullets of commercial interest, *Chelon auratus* and *Chelon saliens*, from the south coast of the Caspian Sea. It was also proposed to assess the relationships between the different pairs of metals in muscle tissue in order to ascertain possible pollution sources in this region.

## 2. Materials and Methods

### 2.1. Study Area and Sampling of Fish Muscle Tissue

**Figure 1:**
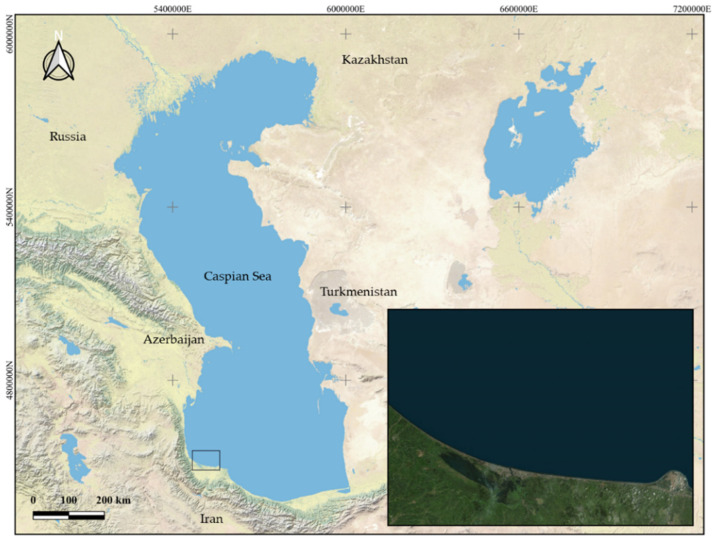
Sampling zone location on the southern coast of the Caspian Sea.

A total of 49 fish from two different species, 20 *C. auratus* and 29 *C. saliens*, were obtained randomly in the southern area of the Caspian Sea, as shown in Figure 1. The fish were caught using nets and were kept on ice until sampling.

**Figure 2:**
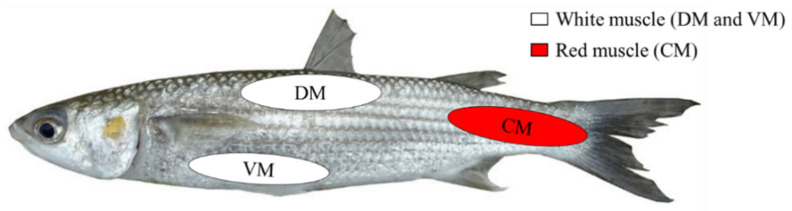
Sampling areas for *C. auratus* and *C. saliens*. Ventral muscle (VM), dorsal muscle (DM) and caudal muscle (CM).

Before collecting the muscle samples, the animals were cleaned with distilled water to remove any dirt or possible external substances that might pollute them. They were then skinned and, from each fish, samples from three different muscle areas were taken: ventral muscle (VM); dorsal muscle (DM) and caudal muscle (CM), as can be observed in Figure 2. The VM and DM samples corresponded to white muscle and the CM sample to red muscle. Muscle samples were taken from each individual fish for the determination of the concentration of each of the metals under consideration, with each sample weighing 1 g. The instrument used to cut the muscle tissue was washed with 1% nitric acid, before each sample was taken. Samples were frozen at −20 °C until their analysis. The sampling was carried out in accordance with the European protection rules for animals used for scientific purposes [28].

### 2.2. Heavy Metals Analysis

The heavy metals analyzed were as follows: As, Cd, Cu, Hg, Ni, Pb, and Zn. For their quantification, an inductively coupled plasma mass spectrometer (ICP-MS) (Agilent 8900-Agilent Technologies, Palo Alto, CA, USA) was used. For their analysis, the preparation of the samples followed a protocol adapted by Bakhshalizadeh et al. [29]. Each sample was homogenized and digested in a digestion solution (15 mL, with 65% nitric acid (HNO_3_) and hydrogen peroxide) and heated on a plate at 200 °C. When the sample reached a volume of 5 mL, the content was decanted into a Falcon tube and diluted in deionized water (Milli-Q Millipore 18.2 MΩcm of resistivity) until reaching 30 mL. The analysis blanks were processed in the same way, and the concentrations were determined using standard solutions prepared in the same acid matrix.

### 2.3. Statistical Analysis

The statistical analysis of the data was performed using SPSS 25 software (IBM, Chicago, IL, USA). The data normality was evaluated using the Kolmogorov–Smirnov test. Due to the non-normality observed in the heavy metals analyzed, the data was converted [30]. The first step consisted of turning the variables into a percentile range, which produced uniformly distributed probabilities. The second step applied the reverse of the first step’s results in order to form a variable that presents normally distributed z scores. With this conversion, we obtained non-dimensional data, permitting us to apply parametric methods. A multifactorial analysis of variance (ANOVA) was performed using both the species and the muscle tissue as factors in order to establish the statistical differences in the concentrations of the different metals. The homoscedasticity of the variances was analyzed using the Levene test. The Tukey test was applied as a post hoc method to discern the different statistics between the three muscle areas. A Pearson correlations matrix was made between the different metals. In all the cases, a value of *p* < 0.05 was taken as being significant.

## 3. Results and Discussion

The mean concentrations and the standard deviations determined for the metals analyzed in the different muscles of *C. auratus* and *C. saliens* are shown in Table 1. Notably the different metal concentrations were highly varied in the different samples. Without taking into account the species and the muscle tissue, the accumulation of the metals followed this order, from the highest to lowest concentration (minimum value to maximum value): Zn (2.03–610.89 µg/g) > Cu (0.66–272.24 µg/g) > Ni (0.009–56.91 µg/g) > Pb (0.009–37.17 µg/g) > Hg (0.002–24.63 µg/g) > As (0.06–6.55 µg/g) > Cd (0.005–3.65 µg/g).

With regard to the heavy metal concentrations in muscles, no significant differences were observed between the different muscles for As (*p* = 0.49), Ni (*p* = 0.19), and Hg (*p* = 0.17). In a study conducted on different fish (*Morone saxatilis* and *Esox Lucius*), lower levels of methyl mercury and higher levels of As were found in the red muscle [23]. Conversely, the Hg concentration in the muscle tissue of the caudal peduncle in *Thunnus orientalis* was found to be slightly higher (6%) than in the rest of the fish’s body [31]. However, there are few works informing on Hg concentrations in different muscle areas in fish, and, although it is important to point out that these are different species in all the examples, the results have not been clarified.

For Cd, the caudal muscle gave a significantly higher concentration (*p* < 0.05) compared to the dorsal muscle. The caudal muscle also had a significantly higher concentrations (*p* < 0.05) of Pb and Cu compared to the dorsal and ventral muscles. Zn presented significant differences (*p* < 0.05) between the three muscles analyzed, with the order from the highest to lowest concentration being: CM > DM > VM. Therefore, the red muscle (CM) had a higher concentration for all of these metals (Cd, Cu, Pb, and Zn). This red muscle is highly vascularized, it makes slow contraction movements, is capable of maintaining the contraction, and has an aerobic metabolism. In comparison, the white muscle, is less vascularized, it contracts rapidly and not for long, and has an anaerobic metabolism [25,26,32,33]. In addition, the red muscle has a larger amount of fat and a smaller amount of protein [23]. These structural and physiological variations could explain the higher concentration of some heavy metals in this muscle type.

Among animal species, fish are a suitable bioindicators of metal pollution due to their position at the top of the food chain in aquatic ecosystems [34]. Although, fish muscles are not an active site for metal accumulation and biotransformation [35], they make up most of the part of fish consumed by humans, so the accumulation of metals in this tissue may signify a risk to human health [36]. Another key point with respect to this is the selection of the species as a bioindicator. The two mullets evaluated in this study are heavily fished in the southern Caspian Sea. Regarding this, from 1996 to 2017, *C. auratus* and *C. saliens* represented the 40.8% and 4.5% of the mean annual catch number in this area, respectively [37]. Thus, the heavy metals exposure through these species is highly common. The European Union only established a maximum heavy metal content in fish for Cd, Hg, and Pb and these are placed at 0.05, 0.5, and 0.2 µg/g wet weight, respectively [38].

For Cd, the majority of the samples analyzed, independently of the muscle tissue examined, exceeded the maximum limit imposed by the EU. Regarding Cd toxicity, it was concluded that CNS is one of the most sensitive parts of biological system that can become easily damaged during the early phase of neonatal development. However, when the Cd exposure is chronic, it may also show adverse effect on the adult brain [39]. In fact, Cd chronic exposure induces molecular mechanisms that are involved in Alzheimer’s disease pathogenesis [40]. Furthermore, it has been shown that environmentally relevant Cd concentrations exposure was sufficient to impair adult hippocampal neurogenesis in mice [41]. Chronic exposure to Cd is also associated with kidney damage. Cd accumulates in the proximal tubule, resulting in a generalized resorptive dysfunction characterized by polyuria and proteinuria [42]. It has been revealed that even relatively low Cd exposure through diets increases the risk of low bone mineral density and osteoporosis-related fractures in elderly men [43].

In the case of Hg, only some samples surpassed the maximum limit established. The concentrations found in our study for this metal generally coincide with those described from the Caspian Sea by other authors, ranging between 0.044 µg/g (mean) in the whole body of *Cyprinus carpio* and 3.5 µg/g in the muscle tissue of *Huso huso* [44]. Such as Cd, the brain is the target organ for Hg. Hg causes displacement of Zn in metalloprotein, which causes damage to neurons. It has been revealed that mercury increases TNF-α levels, which promote neuroinflammation and cellular apoptosis, and cause Parkinson’s disease-like symptoms [45]. However, Hg can impair any organ and lead to malfunctioning of nerves, kidneys, and muscles [15]. Inorganic mercury has been found to affect Ca homeostasis and permeability of the plasma membrane [12]. Moreover, it has been shown that Hg has an endocrine effect because it interacts negatively with dopamine [46].

The maximum limit of Pb was only surpassed in the caudal muscle area samples. Lead toxicity is multifactorial since it directly interrupts the activity of several enzymes, competitively inhibits absorption of important trace minerals, mainly Ca, and deactivates some antioxidants. In fact, similar to Cd and Hg, Pb damages cellular components through oxidative stress [47]. Regarding the effects of Pb exposure on human health, it has been associated with memory reduction, cognitive function loss, increased risk of hemolytic anemia, premature baby birth and low weight at birth, reduced sperm count and decreased libido, increased risk factor of still births and miscarriages, and a reduction in amount of vitamin D in the body [39].

The fact that the concentrations reported went beyond the maximum limits of Cd, Hg, and Pb permitted by European legislation is of great concern. However, other factors should be taken into account, including the amount of fish consumed daily, in order to evaluate the potential risk to human health stemming from a prolonged consumption of polluted fish [48,49].

**Figure 3:**
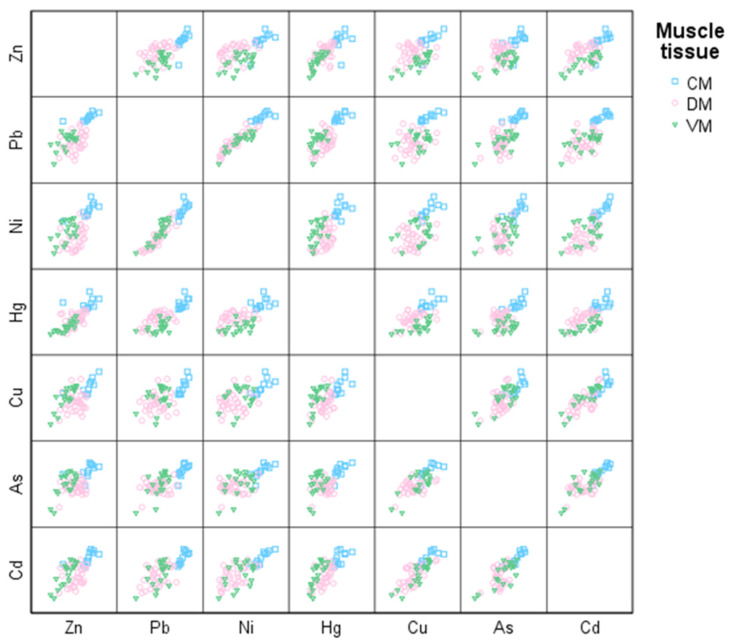
Pearson correlation matrix for the different metals analyzed subdivided into muscle groups.

The Pearson correlations matrix for the different metals shown in Table 2. All the correlations were positive and significant (*p* < 0.05) for the different combinations of metal pairs. We can therefore be certain that, at the same time as the concentration of a particular metal increases, so does the concentration of any other metal. The relationships between metals can be observed graphically and then subdivided into muscle groups, as shown in Figure 3.

These positive relationships in fish muscle tissue are a biomarker of the presence and availability of metals both in the aquatic environment and in the sediments of the region sampled. It also highlights great similarities in their distribution within the environment, and that this distribution is chiefly due to external inputs [50]. In this sense, waste from the oil refinery is probably the main anthropogenic source of pollution [51,52,53], although there are other possible contamination origins, such as industrial discharges or domestic sewage [54]. It has also been seen that, when the relationship is significant, as in our study, it is the sign of a common or similar origin of the pollutants [53,55]. Oil production near the Caspian Sea covers extensive areas of the coastal zone, especially on the south coast [56], which would explain our results. However, the discharge of petroleum products into rivers could be another important source of pollution, since every year 75,000 tons of these products are discharged into the rivers that flow into the Caspian Sea [57].

On the other hand, an analysis of thousands of water samples, collected between 2014 and 2019 in several areas near the coast of Kazakhstan, concluded that the Caspian Sea was “fairly or marginally” polluted, with high levels of Cd and Pb concentrations, and extreme atypical values in the majority of the areas [58]. That coincides with the results obtained in our study, in which high Cd and Pb concentrations were found in the muscle tissue of all the fish analyzed.

## 4. Conclusions

The accumulation of metals in both fish species studied here, *C. auratus* and *C. saliens*, share similar accumulation patterns, and only differ in the accumulation of Cd and Hg, which are higher in *C. saliens*. In the case of the musculature, the caudal (red) muscle presented higher concentrations of Cd, Cu, Pb, and Zn. By contrast, the accumulation of As, Hg, and Ni seemed to be independent of the muscle type.

Heavy metal concentrations of Cd, Hg, and Pb in fish musculature exceed the maximum limits permitted by the EU, which is a matter of particular concern for human health.

The results obtained here can be considered to be a biomarker of the presence and availability of heavy metals, both in the aquatic environment, and in sediments, and is a sign of anthropogenic pollution in the area sampled.

These results emphasize the need to continue to monitor and evaluate the degree of pollution in the Caspian Sea, both in fish and other species and in the environment, as well as recommending prevention measures orientated towards limiting and/or reducing the excessive exposure of the population to heavy metal content.

## Figures and Tables

**Table 1:** Mean values plus minus standard deviations of the metals analyzed in *Chelon auratus* and *Chelon saliens*.

Metal (µg/g)	Fish Species					
	*Chelon auratus*			*Chelon saliens*		
	Muscle					
	CM	VM	DM	CM	VM	DM
As	1.71 ± 2.21	0.65 ± 0.42	0.43 ± 0.14	1.29 ± 1.51	0.68 ± 0.20	0.52 ± 0.15
Cd *	0.45 ± 0.7 ^b^	0.10 ± 0.18 ^a^	0.04 ± 0.04	0.38 ± 0.8 ^b^	0.18 ± 0.19 ^a^	0.05 ± 0.05
Cu	133.53 ± 50.80 ^bc^	28.04 ± 73.84 ^a^	8.60 ± 22.73 ^a^	48.53 ± 49.79 ^bc^	27.62 ± 18.11 ^a^	11.60 ± 26.72 ^a^
Hg *	4.75 ± 8.18	0.03 ± 0.02	0.06 ± 0.03	3.23 ± 5.36	0.05 ± 0.02	0.07 ± 0.03
Ni	10.43 ± 18.33	0.32 ± 0.23	0.30 ± 0.33	5.20 ± 7.62	0.45 ± 0.34	0.44 ± 0.93
Pb	7.31 ± 11.77 ^bc^	0.11 ± 0.08 ^a^	0.16 ± 0.23 ^a^	6.01 ± 7.17 ^bc^	0.14 ± 0.06 ^a^	0.12 ± 0.18 ^a^
Zn	375.16 ± 140.37 ^bc^	7.84 ± 4.51 ^ac^	18.77 ± 5.66 ^bc^	201.38 ± 204.60 ^bc^	17.66 ± 2.28 ^ac^	20.14 ± 20.46 ^ab^

* Significant difference between species (*p* < 0.05). ^a^ Significant difference with CM (*p* < 0.05). ^b^ Significant difference with VM (*p* < 0.05). ^c^ Significant difference with DM (*p* < 0.05).

**Table 2:** Pearson correlation matrix * between the different metals analyzed.

	Zn	Pb	Ni	Hg	Cu	As	Cd
Zn	1	0.669	0.624	0.766	0.569	0.592	0.638
Pb		1	0.374	0.343	0.589	0.417	0.641
Ni			1	0.773	0.611	0.681	0.561
Hg				1	0.604	0.564	0.579
Cu					1	0.763	0.844
As						1	0.699
Cd							1

* All the correlations were significant (*p* < 0.05).

## Data Availability

The data that support the findings of this study are available on request from the corresponding author.
